# A simple method to improve the antibiotic elution profiles from polymethylmethacrylate bone cement spacers by using rapid absorbable sutures

**DOI:** 10.1186/s12891-022-05870-0

**Published:** 2022-10-14

**Authors:** Tzu-Hao Tseng, Chih-Hao Chang, Chien-Lin Chen, Hongsen Chiang, Hao-Ying Hsieh, Jyh-Horng Wang, Tai-Horng Young

**Affiliations:** 1 Department of Biomedical Engineering, National Taiwan University, No.1 Jen Ai road section 1, 10002 Taipei, Taiwan; 2 Department of Orthopaedic Surgery, National Taiwan University Hospital, 7 Chungsan South Road, 10002 Taipei, Taiwan; 3 Department of Orthopaedic Surgery, National Taiwan University Hospital Jin-Shan Branch, New Taipei City, Taiwan; 4 Department of Biomedical Engineering, National Taiwan University Hospital, Taipei City, Taiwan

**Keywords:** PMMA, Cement, Release, Infection, Antibiotics

## Abstract

**Objective:**

Antibiotic-loaded bone cement beads and spacers have been widely used for orthopaedic infection. Poor antibiotic elution is not capable of eradicating microbial pathogens and could lead to treatment failure. The elution profiles differ among different cement formulations. Although Simplex P cement has the least release amount, it is widely used due to its ready availability. Previous methods aiming to improve the elution profiles were not translated well to clinical practice. We sought to address this by using easily available materials to improve the elution profile of antibiotics from PMMA, which allows clinicians to implement the method intraoperatively.

**Methods:**

Vancomycin was mixed with Simplex P cement. We used Vicryl Rapide sutures to fabricate sustained-release cement beads by repetitively passing the sutures through the beads and/or mixing suture segments into the cement formulation. Vancomycin elution was measured for 49 days. The mechanism of antibiotic release was observed with gross appearance and scanning electron microscopic images. The antimicrobial activities against MRSA were tested using an agar disk diffusion bioassay.

**Results:**

Passing Vicryl Rapide sutures through cement beads significantly improved the elution profiles in the 7-week period. The increased ratios were 9.0% on the first day and 118.0% from the 2nd day to the 49th day. Addition of suture segments did not increase release amount. The Vicryl Rapide sutures completely degraded at the periphery and partially degraded at the center. The antibiotic particles were released around the suture, while antibiotic particles kept densely entrapped in the control group. The antimicrobial activities were stronger in passing suture groups.

**Conclusion:**

Passing fast absorbable sutures through PMMA cement is a feasible method to fabricate sustained-release antibiotic bone cement. Intra-cement tunnels can be formed, and the effect can last for at least 7 weeks. It is suitable for a temporary spacer between two stages of a revision surgery.

## Introduction

Antibiotic-loaded bone cement spacers and beads have been widely used for the treatment of orthopaedic infections, including prosthetic joint infection [1–3], infected non-union [4], and chronic osteomyelitis [5]. In two-stage surgeries, high-dose antibiotic-loaded polymethylmethacrylate (PMMA) cement beads or spacers are implanted after hardware removal and surgical debridement in the first stage. PMMA cement serves as a local antibiotic carrier to achieve local antibiotic concentrations that exceed the minimal inhibitory concentrations. PMMA is usually placed in position for 6–8 weeks before the final prosthesis, or internal fixators are implanted [3, 6]. Therefore, the elution profiles of antibiotics during this period are important. However, the total release percentage of commonly used antibiotics, such as vancomycin, cefazolin and gentamicin, is less than 10% [7–9]. The elution profiles differ among different cement formulations. Palacos cement and Copal cement have been shown to have the highest rates of elution or effectiveness in preventing biofilm formation, whereas Stryker Surgical Simplex P cement has the lowest rates [10, 11]. A previous clinical study showed that the in vivo vancomycin concentration released from Simplex P cement spacer became undetectable on the 7th day after implantation in 40% of patients [12]. However, not all types of cement are available to clinicians. Therefore, the development of practical methods to improve elution profiles is important.

Factors affecting antibiotic elution include antibiotic loading dose, physicochemical properties of antibiotics, mixing methods of antibiotics, cement porosity, and cement composition [13]. Various methods have been developed to improve antibiotic elution profiles, and the creation of high porosity has been a major concept in many studies [13–23]. The incorporation of particles made of various materials, such as gelatin [15], poly(lactic-co-glycolic) acid (PLGA) [22, 23], α-tricalcium phosphate [16], calcium sulphate [18] and antibiotic-loaded nano-sized liposomes [24] as poragens into PMMA cement is most commonly used. These particles can significantly increase the porosity and its interconnectivity of PMMA spacers, facilitating efficient antibiotic release [13]. Another strategy is the use of other biodegradable materials instead of PMMA as antibiotic carriers, such as calcium sulfate [25, 26], PLGA [13], and hydroxyapatite [27]. However, most of these methods are still in the experimental stage. Although there have been some commercialized products, such as Stimulan calcium sulfate beads [25, 26], they are not widely available worldwide. Furthermore, biodegradable carriers cannot serve as temporary spacers during two-stage revision arthroplasty. Therefore, there is a large gap between laboratory and clinical practices, and antibiotic-loaded PMMA cement remains the most popular method.

This study aimed to use easily available materials to improve the elution profile of antibiotics from PMMA, which allows clinicians to implement the method intraoperatively. We hypothesized that biodegradable sutures passing through PMMA could leave tunnels to facilitate antibiotic release after the sutures are hydrolyzed.

## Methods

### Fabrication of antibiotic-loaded PMMA

Having the less favorable antibiotic elution data, Stryker Surgical Simplex P [Stryker, Kalamazoo, MI] was the target PMMA product in this research. It is a commercial bone cement widely used worldwide. The chemical composition of Simplex P bone cement is shown in Table 1. The P/L ratio is 2.11. The viscosity of Simplex P at room temperature is classified as medium viscosity [28]. Vancomycin was chosen as the target antibiotic because of its common use in clinical practice and its activity against methicillin-resistant Staphylococcus aureus (MRSA), which is one of the most common pathogens causing osteomyelitis and implant-related infections [29, 30]. The Vicryl Rapide suture (Ethicon, USA) was chosen because of its fast absorption with total hydrolysis at 42 days, which is approximately 6–8 weeks between the two stages of revision arthroplasty. The Vicryl Rapide suture is composed of a copolymer made from 90% glycolide and 10% L-lactide.

**Table 1:** Chemical composition of Simplex P bone cement

**40 g of powder**	
Poly (methymethacrylate)	15%
Poly (methylacrylate-styrene)	73.5%
Barium sulfate	10%
Benzyl peroxide	1.5%
**20mL of liquid (19 g)**	
Methy methacrylate	97.45%
N,N-Dimethyl-p-toluidine	2.55%
Hydroquinone	80ppm

The Simplex P bone cement consists of 40 g of power and 19 g of fluid. All values are given as wt/wt

We speculated that passing sutures through the bone cement and adding suture segments to the bone cement might improve antibiotic elution. Passing biodegradable sutures through PMMA ensures the exposure on PMMA surface at both ends of the suture. It may leave tunnels to facilitate antibiotic release after hydrolysis. Suture segments might act as poragens to create high porosity after hydrolysis of the segments. In previous studies, the addition of biodegradable particles as poragens effectively created a high porosity [15, 22, 23].

**Fig. 1:**
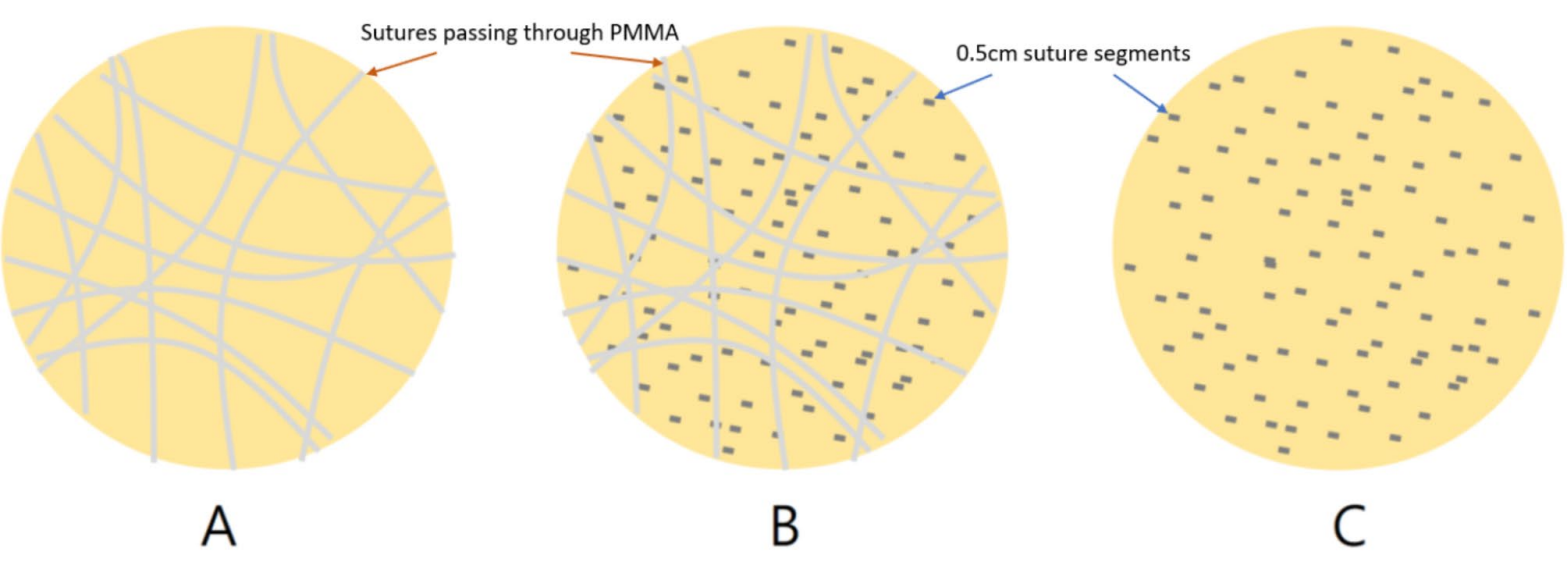
The experimental groups: (A) Passing suture group. (B) Passing suture + segment group. (C) Segment group. Light gray lines: sutures passed through cement beads. Dark gray segments: 0.5 cm suture segments

There was one control group (no suture) and three experimental groups: (1) passing suture group, (2) passing suture + segment group, and (3) segment group (Fig. 1). Briefly, 2 g of vancomycin powder (Sigma, USA) was added to 40 g of PMMA powder. For the “segment group” and the “passing suture + segment group”, 180 cm (two sachets) of 2 − 0 (0.3 mm in diameter) undyed Vicryl Rapide sutures were cut into 0.5 cm segments and mixed with the PMMA/vancomycin powder in this step. The sutures were 0.19 gm. The percentage of additive by weight was 0.48% for this modification. The mixed powders were hand-stirred for 2 min before the addition of methylmethacrylate (MMA) liquid. Spherical beads with a diameter of 20 mm were created using silicone molds. For the “passing suture group” and the “passing suture + segment group”, we passed 50 cm of Vicryl Rapide sutures repetitively through each bead during the dough phase. Beads without Vicryl Rapide sutures served as the control group. The temperatures of the cement beads were measured every 30 s until the temperature decreased below 50°C.

### Antibiotic elution profile

Elution profile analysis was performed according to previous studies, with minor modifications [11]. Briefly, two beads were placed in a 50-mL flask with 15 mL of sterile phosphate-buffered saline (PBS), a volume sufficient to completely submerge both beads. Samples were stored at 37°C for 49 days, with PBS removed at 24-hour intervals for 10 days, at 3-day intervals for 12 days, at 5-day intervals for 20 days, and at 7-day intervals for the last 7 days. The vancomycin concentration of each sample was then measured by UV–vis spectroscopy using a SpectraMax Plus-384 spectrophotometer (Molecular Devices, Sunnyvale, CA, USA). [31]. The concentration was determined by measuring absorbance at 282 nm. The absorbance of monomers, glycolic acid, and lactic acid released after hydrolysis of Vicryl Rapide sutures was extremely low at 282 nm [32, 33]. The experiments were performed in triplicate.

### In vitro cytotoxicity assay

We used a human osteosarcoma cell line, MG-63 (from European Collection of Authenticated Cell Culture, ECACC code: 86,051,601), to test the cell viability. Briefly, MG-63 cells were seeded to 96-well plates at a density of 1 × 10^4^ cells/well. After incubation for 24 h, the cells were incubated in fresh media supplemented with 10% PMMA immersed PBS at 1st, 10th, 19th, 32nd and 49th days. After 24 h, the cells were placed in fresh medium containing 3-(4,5-dimethylthiazol-2-yl)-2,5- diphenyltetrazolium bromide (MTT, Sigma, St. Louis, MO, USA) and incubated at 37 ◦C for 3 h. The absorbance was measured at 570 nm with UV–vis spectroscopy using a SpectraMax Plus-384 spectrophotometer (Molecular Devices, Sunnyvale, CA, USA). The cell viability in each group was calculated and expressed as a percentage relative to that of the control group.

### Bioassay of antibiotic activity

**Fig. 2:**
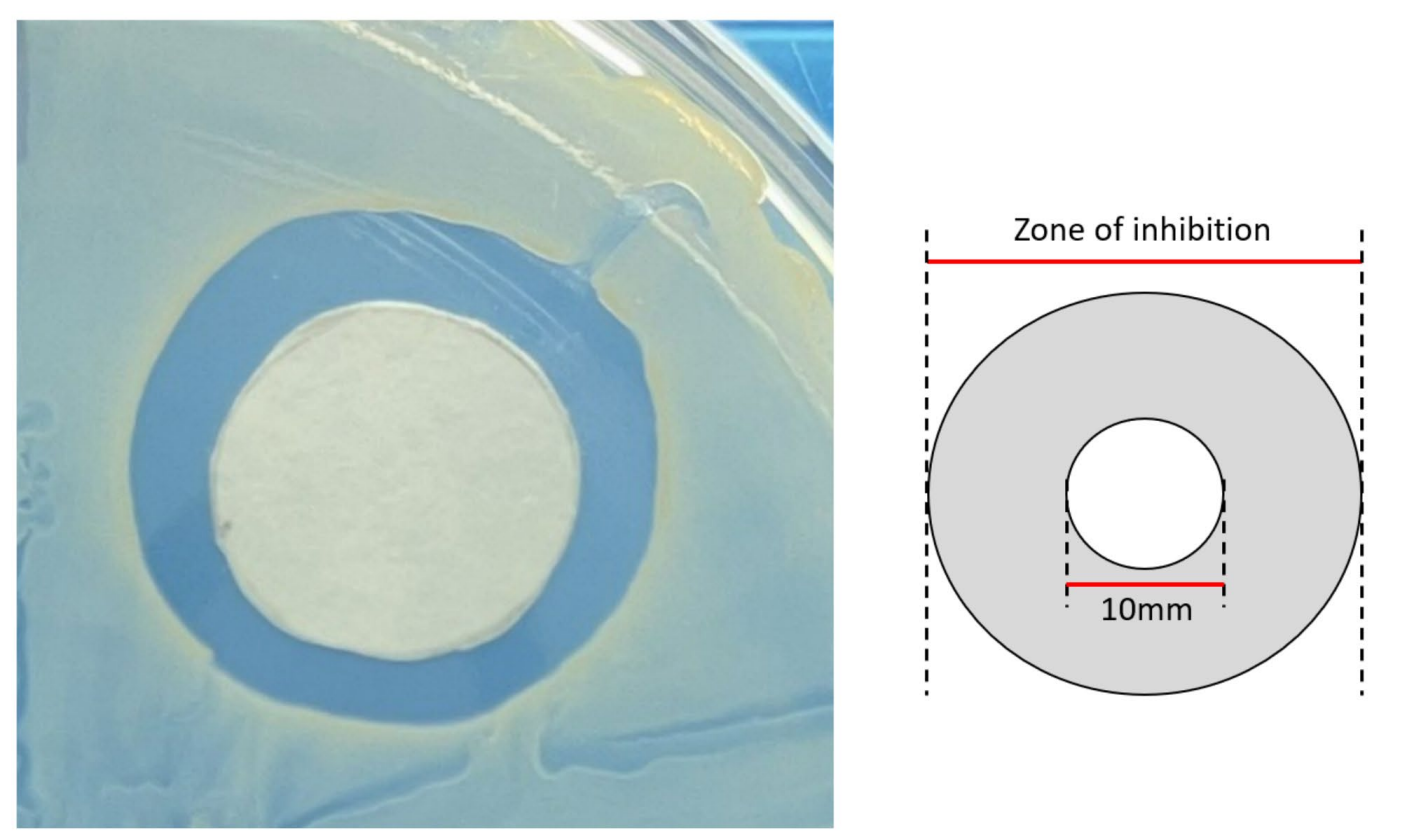
A. The presentative photo of agar disk diffusion bioassay (the result of the 1st-day sample for the passing suture group). B. The illustration of measurement of ZOI.

The antimicrobial activity of bead-immersed PBS collected on the 1st, 10th, 19th, 32nd and 49th days was estimated using an agar disk diffusion bioassay. Briefly, 10-mm antibiotic discs were loaded with 30µL of the PBS samples. The discs were subsequently placed on Mueller-Hinton agar plates (Sigma, St. Louis, MO, USA) inoculated with methicillin-resistant Staphylococcus aureus at 37°C. The zone of inhibition (ZOI) was measured after 24 h to compare the antimicrobial activity between each group (Fig. 2).

### Gross appearance and microarchitecture of PMMA/sutures

After immersion in PBS, the PMMA beads were fractured using osteotomes. The gross appearance was observed to determine whether the sutures were completely hydrolyzed. The fracture surfaces were studied using a JSM-7600 F scanning electron microscope (SEM)(JEOL, Japan). An energy dispersive X-ray spectroscopy (EDS) was also used to visualize and analyze the surface features. The energy of the electron beam was 10 kV. To ensure high quality images, the samples were sputter-coated with platinum using a JFC 1600 auto-fine coater (JEOL, Japan).

### Statistical analysis

All statistical analyses were performed using Real Statistics Resource Pack software (release 8.0) on a Microsoft Windows-based computer. The antibiotic release amounts in the four groups at each time point were compared using the Friedman test. Post-hoc analysis with the Nemenyi test was conducted to identify which pairwise groups had a significant difference. Statistical significance was set at a p < 0.05.

## Results

**Fig. 3:**
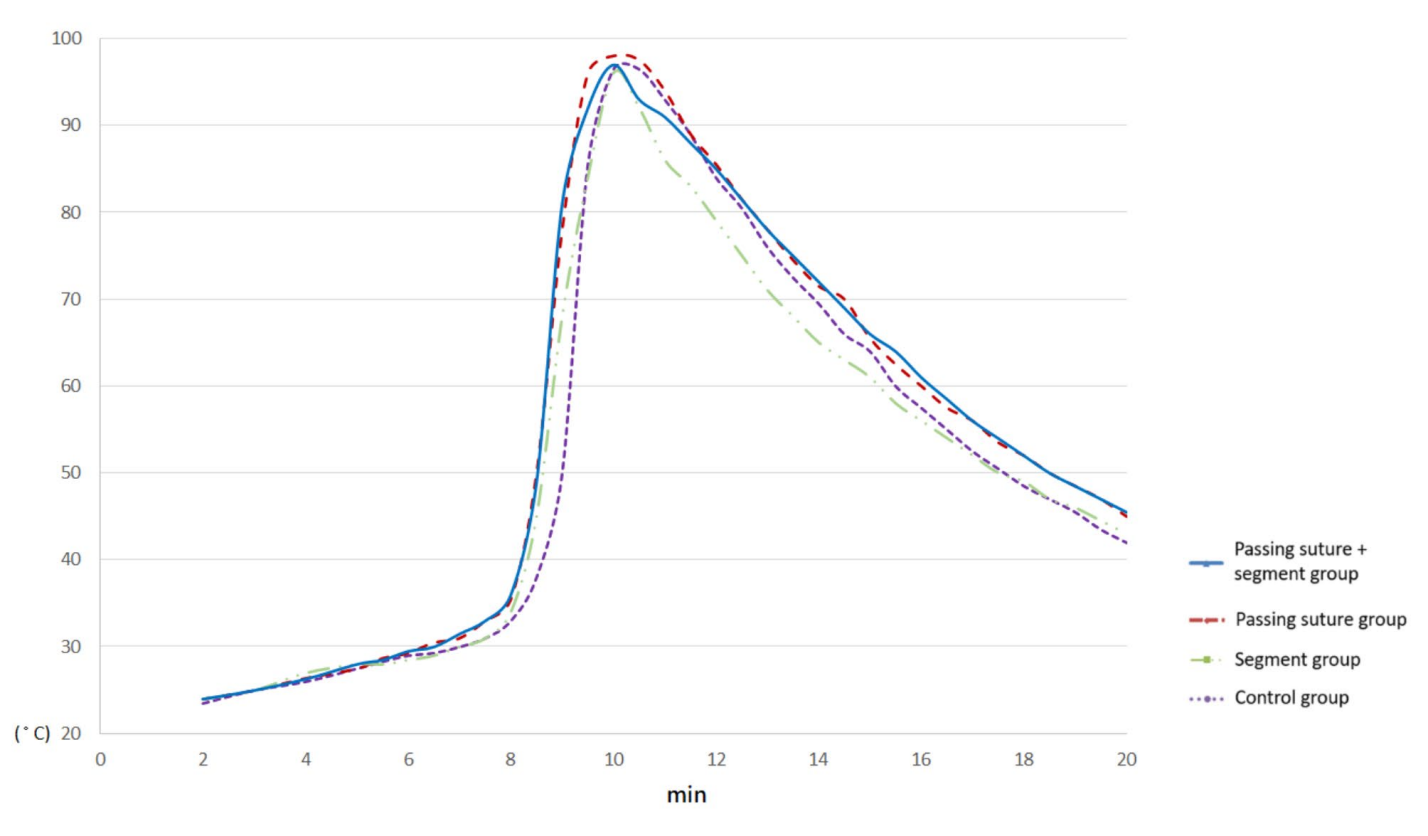
The polymerization temperature of bone cement

**Fig. 4:**
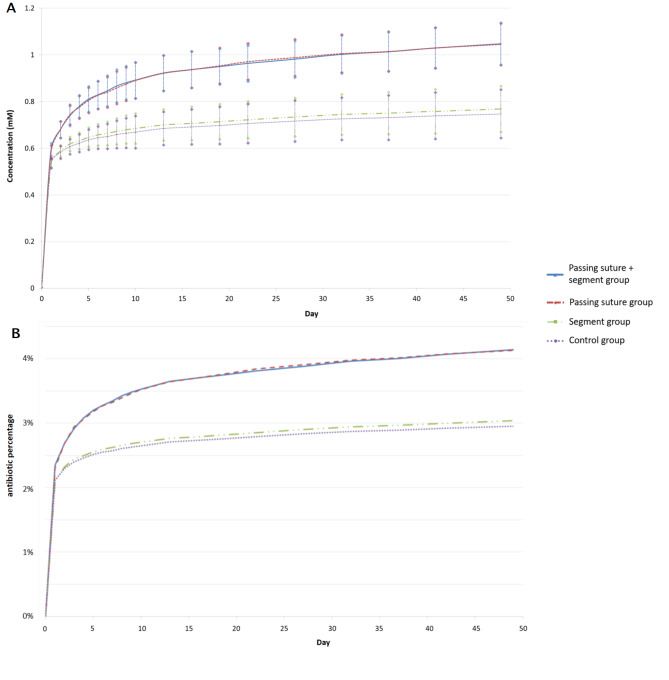
The cumulative antibiotics from the PMMA cement. Passing rapid absorbable sutures increased the release amount

The polymerization temperature is shown in Fig. 3. The peak temperature was comparable between each group (*P* = 0.77). The curing time of the control group was 8 min 52 s ± 15 s. Compared with the control group, the mean curing time was 22 s shorter in the “passing suture group” (*P* = 0.007) and 20 s shorter in the “passing suture + segment group” (*P* = 0.006). The mean value of the cumulative antibiotic release from the three experimental groups and the control group is shown in Fig. 4. The amount released among the four groups was significantly different (*P* < 0.001 in the Friedman Test). The post-hoc analysis showed that the “passing suture group” and the “passing suture + segment group” released more antibiotics than the “segment group” and the control group (*P* < 0.001). The addition of suture segments did not increase the antibiotic release amount (*P* > 0.99 for the “passing suture group” vs. the “passing suture + segment group”, and *P* = 0.82 for the “segment group” vs. the control group). A burst release was observed in each group on the first day. Compared with the control group, the total release amount from the “passing suture group” increased by 39.9%. The increased ratios were 9.0% on the first day and 118.0% from the 2nd day to the 49th day.

**Fig. 5:**
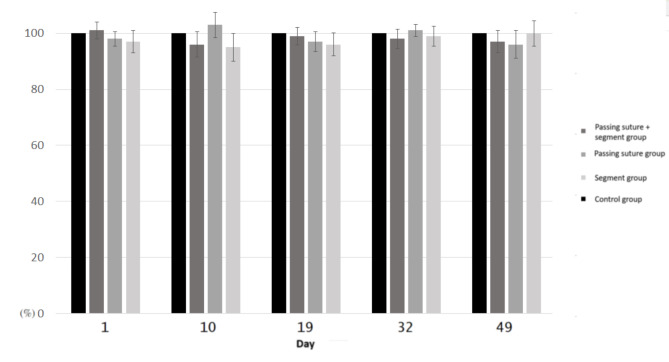
The results of cytotoxicity assay. The cell viability was comparable between each group

**Fig. 6:**
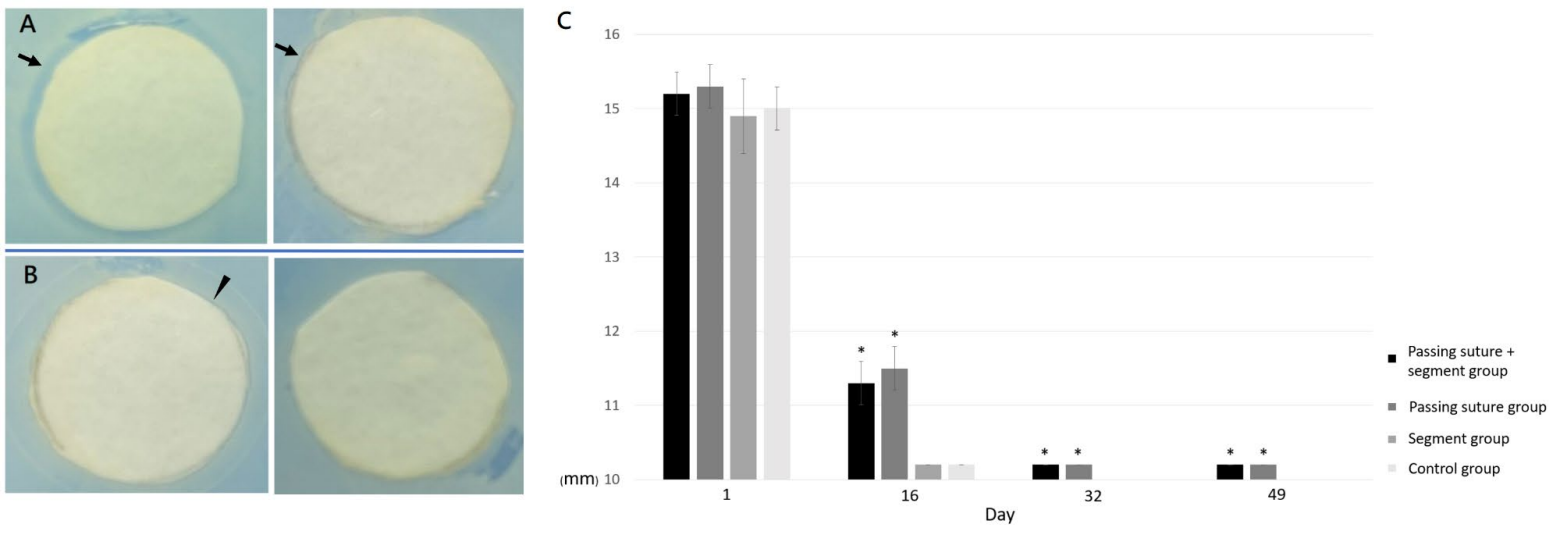
The results of bioassay of antibiotic activity. (A) The ZOI was larger in the “passing suture group” and “passing suture + segment group” (arrow in the left photo) for the 16th-day samples, whereas the ZOI was narrow in the control group and the “segment group” (arrow in the right photo). (B) The ZOI was invisible in the control group and the “segment group” (right photo), whereas there was still a narrow ZOI in the “passing suture group” and “passing suture + segment group” (arrowhead in the left photo) for the 32nd and 49th-day samples. (C) The ZOI was comparable between each group for 1st-day samples and significantly different for 16th-day, 32nd-day and 49th-day samples. * *P* < 0.05 compared to the control group

The cell viability of each group was shown in Fig. 5. Passing sutures and the addition of suture segments did not affect cell viability (*P* > 0.05 in the Friedman Test). The antimicrobial activities of the bead-immersed PBS were shown in Fig. 6. The ZOI of the 1st-day PBS-loaded disc was comparable between each group. The ZOI was larger in “passing suture group” and the “passing suture + segment group” for the 16th-day, 32nd-day and 49th-day PBS-loaded discs.

**Fig. 7:**
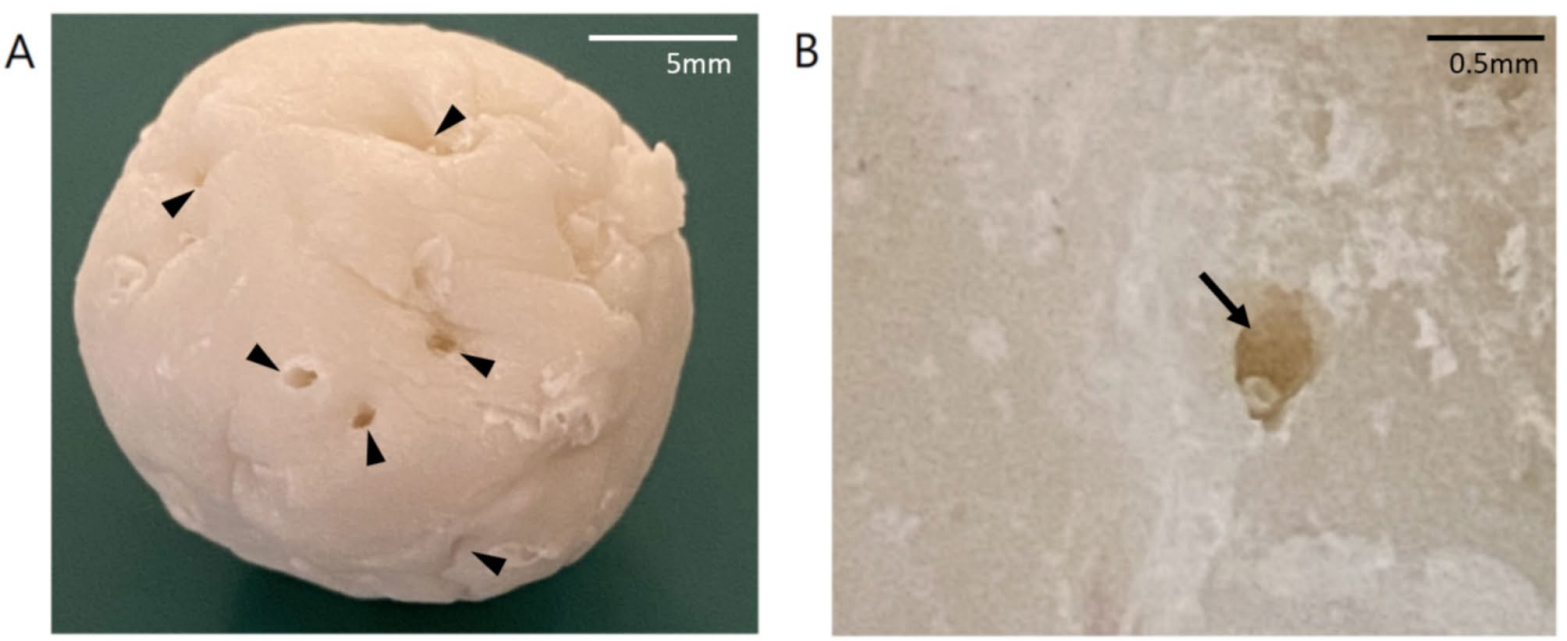
The gross appearance of the cement bead after PBS immersion. (A) The sutures at the most peripheral area were completely hydrolyzed (arrowhead). (B) The sutures at the fracture surface near the bead center were partially hydrolyzed, and visible tunnel space appeared around the suture. (arrow)

**Fig. 8:**
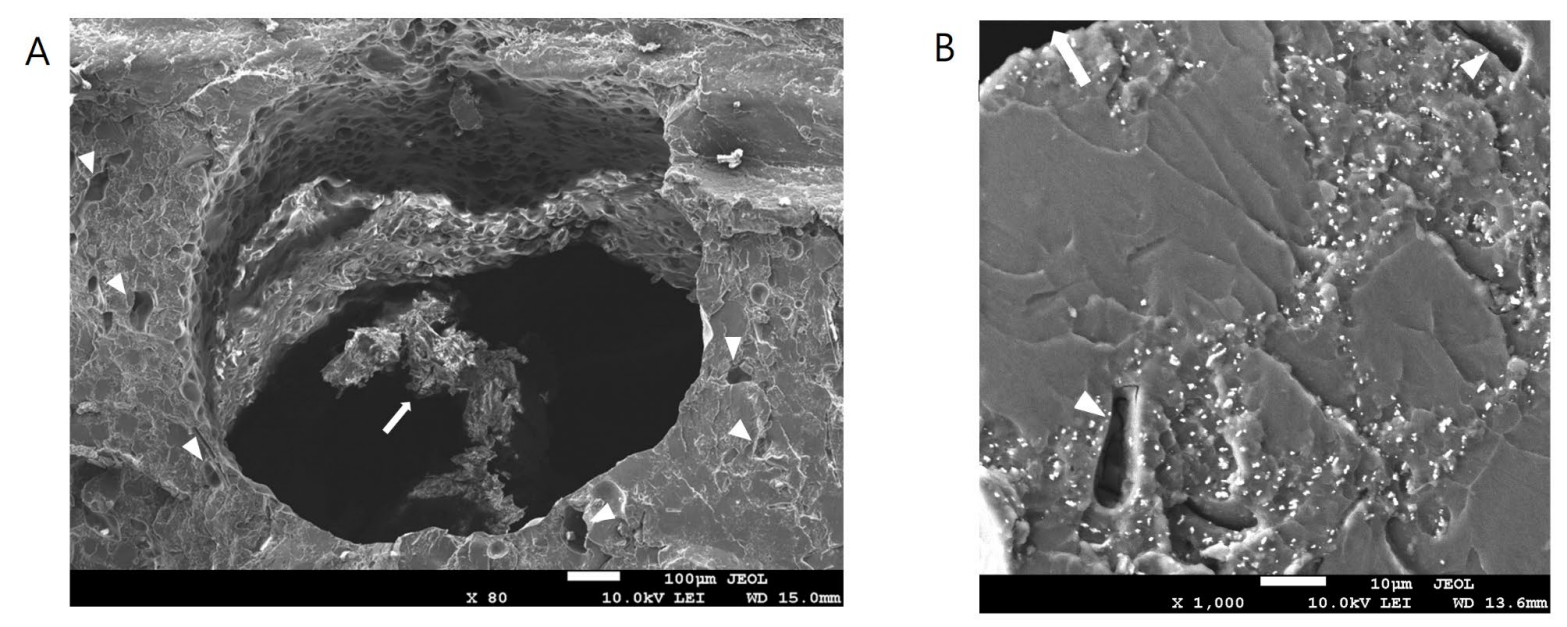
SEM image of the suture passed cement beads (outer half) after PBS immersion. (A) Sutures were almost completely hydrolyzed at the outer half of the bead. Arrow: the remaining suture. Arrowheads: empty nest holes after antibiotics were released. (B) High magnification image (1000x). Arrow: the tunnel space. Arrowheads: empty nest holes after antibiotics were released

**Fig. 9:**
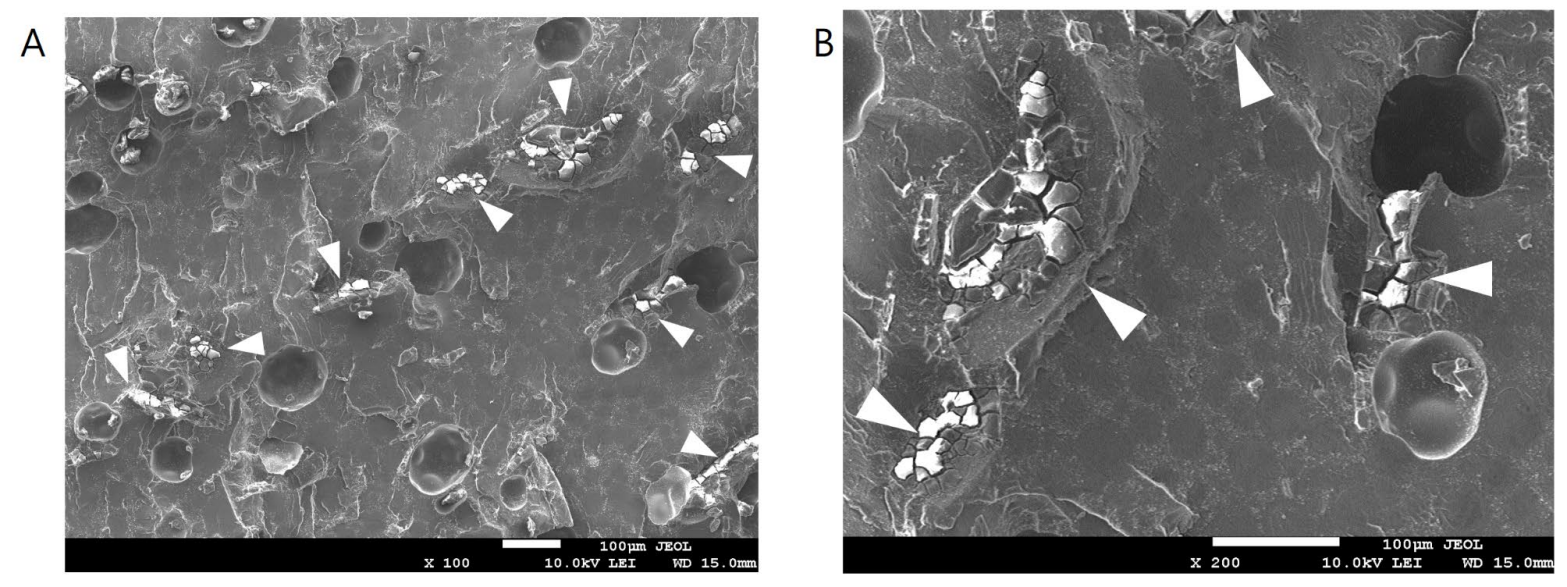
SEM image of the cement beads without suture (control group). Arrowheads: densely entrapped antibiotic particles

**Fig. 10:**
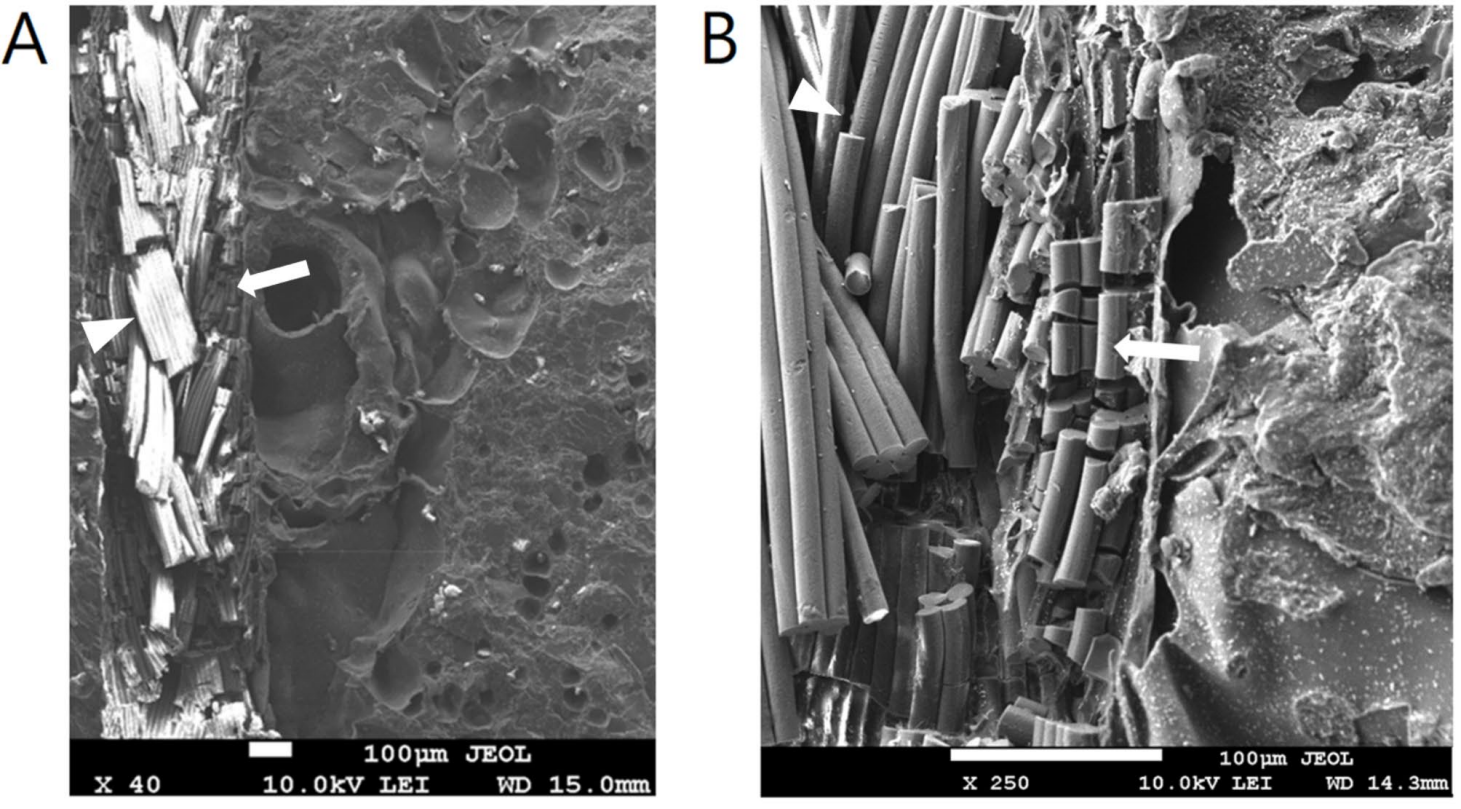
SEM image of the suture passed cement beads (near the bead center) after PBS immersion. (A) Sutures were partially hydrolyzed near the bead center. (B) High magnification image (250x). Arrow: the peripheral filaments degraded to much shorter segments. Arrowheads: the core filaments

Regarding gross appearance, the sutures in the most peripheral area were completely hydrolyzed (Fig. 7 A), whereas the sutures near the center of the beads were partially hydrolyzed. The sutures became much thinner and fragile, leaving an obviously visible tunnel space around them (Fig. 7B). Analysis of the SEM images showed that the sutures were almost completely hydrolyzed in the outer half of the beads (Fig. 8). Antibiotic particles around the suture tunnel were absent, and their nest holes were left empty. The nest holes were the spaces previously embedded with antibiotic particles. In contrast to the round pores in the PMMA cement, their shape was irregular and consistent with that of the antibiotic particles in Fig. 9. In accordance with the gross appearance, the sutures at the bead center were partially hydrolyzed. The peripheral filaments degraded to segments that were much shorter than the core filaments (Fig. 10). These results indicate that the degradation began from the periphery of the multifilament sutures, and the tunnel space appeared and gradually enlarged. In the control group, numerous antibiotic particles were still densely entrapped in the outer half and center of the cement beads (Fig. 9).

## Discussion

The most important contribution of this study is that we successfully improved the vancomycin elution profile by simply passing fast absorbable sutures through antibiotic-loaded PMMA. Because the Vicryl Rapide suture is a commonly used commercialized product [34, 35], its safety has been well established. Furthermore, orthopaedic surgeons are familiar with the skills required to pass sutures through PMMA beads or spacers in the dough phase. Therefore, this method is feasible and easily operable in the clinical practice.

Antibiotic release from PMMA cement has been previously reported. The Simplex P cement has the lowest release amount among various formulations. However, it is widely used to fabricate cement spacers owing to its availability [12, 36]. In consistent with previous in vitro studies [7], the present results showed that the cumulative vancomycin release from plain Simplex P cement was less than 3%. In vivo vancomycin concentration using Simplex P cement spacers could become undetectable in the early period [12], which may be a cause of treatment failure [36]. Therefore, improving the elution profile of antibiotics is important in clinical practice. Regardless of the cement formulation, there is always a burst of antibiotic release on the first day [31]. The release amount subsequently drops rapidly to an extremely low level, and most of the antibiotics remain entrapped inside the PMMA cement. Although various methods have been developed to facilitate antibiotic release, many would increase the burst amount on the first day [13, 15]. The burst release of antibiotics can cause transient high serum antibiotic concentrations, resulting in acute kidney injury [37–39]. In contrast, the current method only increased a small amount of vancomycin (9%) on the first day, whereas there was a more than two-fold increase in the release amount in the following 7 weeks. Since cement spacers or beads are usually retained in position for 6–8 weeks in two-stage revision surgeries [3, 6], the release in the late period is important to ensure successful eradication of the pathogen. In the current study, the absorption of biodegradable sutures began from the periphery to the center of the spherical beads. The antibiotic particles observed with SEM were absent around the absorbed sutures, whereas numerous particles were still densely entrapped in the control group. Therefore, it could be speculated that the antibiotics were released after the tunnel space appeared. In addition to passing sutures through bone cement, we also mixed 0.5 cm suture segments into the PMMA powder to simulate previous methods [15, 17, 22]. However, the addition of suture segments did not result in improved elution profiles. The possible reason was that the number of segments may be insufficient because there were only 360 suture segments in 40 g of PMMA. Insufficient segments might result in decreased suture exposure on the bead surface and poor connection between suture segments. Because PMMA is highly hydrophobic, decreased suture exposure and poor inter-segment connection would prevent hydrolysis of the suture segments trapped inside the bone cement. Therefore, these segments could not act as poragens well. A previous study using gelatin particles as poragens showed that a higher number of particles results in higher porosity [15]. These suture segments were cut from two sachets of Vicryl Rapide sutures. It would be less cost-effective to have enough segments cut from more sachets of sutures. Since this study aimed to develop a practical method, we did not add more segments.

Regarding the sustained release of antibiotics from PMMA, promising results have been reported in previous studies [13–18, 21, 22, 24, 40, 41]. One major concept of many studies was the creation of high porosity and interconnectivity of the bone cement [13, 15–18, 21, 22, 24, 40]. For example, the addition of 200–400 μm gelatin particles as poragens could increase porosity, resulting in an increase in the amount and rate of drug release. It also made the mechanical properties of PMMA cement more comparable to those of cancellous bone [15]. Moreover, the incorporation of many other degradable materials, such as α-tricalcium phosphate [16], calcium phosphate [18] and PLGA [23, 42] has yielded good results. Previous reports have shown that the controlled and gradual release of antibiotics after the addition of poragens can last for a long period ranging from 4 to 8 weeks [13, 18]. Recently, nanotechnology-based carriers have been developed to improve the release kinetics of antibiotics from PMMA [43, 44]. These nano-carriers include liposomes [24], mesoporous silica [45, 46], hydroxyapatite nano-rods [47], magnetic nanoparticles [48], titanium dioxide (TiO2) nanotubes [49–51], carbon nanotubes [52] and clay nanotubes [53]. The addition of nanoparticles could provide larger surface area, increase local antibiotic concentration, and exert synergistic antibiotic effect by carrying multiple antibiotics [47]. For example, the total release of vancomycin from TiO_2_ nanotubes functionalized Simplex-P bone cements could be more than 50% of the total amount. The extended release of vancomycin could last for at least 80 days. Besides, the mechanical properties were well preserved after incorporation of the TiO_2_ nanotubes [49]. Similarly, the nano-sized liposomal drug delivery system for PMMA bone cement increased release of gentamicin from less than 10% to more than 20% of total antibiotics in 60 days [24]. In addition to the concept of sustained antibiotic release, a refillable antibiotic system composed of insoluble cyclodextrin (CD) microparticles and PMMA has been developed [41, 54, 55]. This delivery system not only allows consistent release of antibiotics but also enables antibiotic refilling through affinity-based interactions. The duration of the consistent release could be more than 60 days and the refilling capacity could provide an additional period of antimicrobial activity for about 30 days [54], which allows complete eradication of pathogenic bacteria.

Apart from the incorporation of the above materials, another strategy is to change the composition of the PMMA cement [14]. A reduction in the L/P ratio and an increase in the radiopacifier ratio can lead to increased porosity, pore diameter and antibiotic elution. The cumulative antibiotic release from PMMA at 70% L/P ratio was more than 70% of the total antibiotics, while PMMA at 100% L/P ratio released less than 20% of antibiotics [14]. However, the burst release from PMMA at 70% L/P ratio within the first day was also five times greater than that from PMMA at 100% L/P ratio, which could lead to antibiotic toxicities. Aeration is another possible method for creating a high porosity [20]. A prolonged aeration time could improve the open-pore structure and increase the average pore size. Although antimicrobial activity has been tested to prove its effectiveness, the released antibiotics have not been quantified. Further studies are required to determine the optimal amount and rate of release using aeration. Recently, various biodegradable antibiotic carriers have been fabricated using calcium phosphates, calcium sulfate hemihydrate, bioactive glass, synthetic polymers and composites [19, 26, 56–60]. The strengths of these biodegradable carriers include better osteogenesis, sufficient antibiotic release and no need of surgical removal. A well-designed biodegradable carrier can provide a sufficient antibiotic concentration in the bone for at least 7 weeks, which can eradicate bacteria in animal models of chronic osteomyelitis [19]. Nevertheless, these carriers cannot serve as appropriate spacers because of their biodegradable nature. Bone cement made of other undegradable materials, such as silorane-based biomaterial [61], may be a solution. Silorane-based cements were initially developed for the use in dentistry. Compared to the PMMA cement, the silorane-based bone cement could release more rifampin and vancomycin within two weeks. Furthermore, the mechanical properties of silorane-based bone cement were not affected by the addition of antibiotics. However, to the best of our knowledge, most of these experimental results have not been translated well into clinical use. The causes of this phenomenon are multifactorial, including unproven safety, high costs, and the complex commercialization process of a new product. Therefore, antibiotic-loaded PMMA spacers without the addition of any other materials remains the most commonly used method. Compared to the results of above studies, the increase amount of vancomycin by using rapidly absorbable sutures was much less. We speculate that the surface area created by the suture tunnels was much smaller than that created by the poragens in previous studies. However, the materials in the present study are easily available to orthopaedic surgeons. Therefore, this method appears to be a transitional solution for improving the elution of antibiotics.

Because the addition of other materials may disturb the polymerization reaction and increase the release of the toxic MMA monomer, cytocompatibility test is essential to determine the safety. There are various methods, such as MTT assay, lactate dehydrogenase assay and cell counting kit-8 assay, to test the cytotoxicity. In previous studies, MTT cellular viability assay has been the most common method to test the cytotoxicity of bone cements [18, 19, 48, 52]. The cell viability was not affected by the addition of magnetic nanoparticles [48], carbon nanotubes [52], calcium phosphate [18], and gelatin [15] into PMMA. In the present study, the cell viability did not decrease after the incorporation of Vicryl Rapide sutures. Therefore, this is a safe method to improve the antibiotic elution from PMMA cement spacers.

This study has several limitations. First, we could only confirm the elution profile of vancomycin. Clinicians can use different antibiotics. Vancomycin is one of the most common antibiotics used in cement spacers. Further studies are necessary to determine the effects of other antibiotics. Second, to simulate the general condition, we used an intermediate size of spherical cement beads between the larger cement spacers and smaller beads. It remains unclear whether different sizes or shapes would have different effects. Nevertheless, the rapid absorbable sutures are supposed to degrade in a similar way, which could facilitate antibiotic release as well. Finally, we did not test the mechanical properties of the PMMA cement. Bone cement with mechanical properties close to those of the surrounding cancellous bone may improve prosthesis longevity [15]. Because passing sutures is not possible for definite fixation of the final prosthesis, this method is suitable for temporary spacers between the two stages of a revision surgery only. Because the spacers are removed in the second stage, their mechanical strength appears to be of minimal importance.

In conclusion, passing fast absorbable sutures repetitively through PMMA cement is a feasible method to fabricate sustained-release antibiotic-loaded bone cement. Intra-cement tunnels can be formed after suture degradation. The effect can last for at least 7 weeks, which is suitable for a temporary spacer between the two stages of a revision surgery.

## Data Availability

The data and material in this research have been all described in the article.

## Abbreviations

EDS: energy dispersive X-ray spectroscopy
MMA: methylmethacrylate
MRSA: methicillin-resistant Staphylococcus aureus
PBS: phosphate buffered saline
PLGA: poly(lactic-co-glycolic) acid
PMMA: Polymethylmethacrylate
SEM: scanning electron microscope
ZOI: zone of inhibition (ZOI)
